# Performance of a hermetic device and neem (*Azadirachta indica*) in storing wheat seed: Evidence from participatory household trials in central Bangladesh

**DOI:** 10.1016/j.jspr.2022.102024

**Published:** 2022-12

**Authors:** Timothy J. Krupnik, Khaled Hossain, Jagadish Timsina, Md. Mohi Uddin, Md. Elahi Baksh, Md. Zakaria Hasan, Mahesh K. Gathala

**Affiliations:** a International Maize and Wheat Improvement Centre (CIMMYT), Sustainable Agrifood Systems Program, Gulshan 2, Dhaka, 1212, Bangladesh; b Institute for Study and Development Worldwide, Homebush West, Sydney, and Global Evergreening Alliance, Burwood, Melbourne, Australia; c Bangladesh Agricultural Research Institute (BARI), Debigonj, Panchagarh, Bangladesh; d Independent Consultant, Dhaka, Bangladesh

**Keywords:** Hermetic storage, Wheat (*Triticum aestivum)*, Household trials, Participatory action research, neem (*Azadirachta indica*), Gender, Arthropod pests and diseases

## Abstract

Smallholder farmers in Bangladesh often use low-density polyethylene (LDPE) bags contained within woven polypropylene bags to store wheat seed during the summer monsoon that precedes winter season planting. High humidity and temperature during this period can encourage increased seed moisture and pests, thereby lowering seed quality. Following a farm household survey conducted to inform trial design, eighty farmers were engaged in an action research process in which they participated in designing and conducting trials comparing traditional and alternative seed storage methods over 30 weeks. Factorial treatments included comparison of hermetic SuperGrainbags® (Premium RZ) against LDPE bags, both with and without the addition of dried neem tree leaves (*Azadirachta indica*). SuperGrainbags® were more effective in maintaining seed moisture at acceptable levels close to pre-storage conditions than LDPE bags. Both seed germination and seedling coleoptile length were significantly greater in hermetic than LDPE bags. Neem had no effect on seed moisture, germination, or coleoptile length. SuperGrainbags® were also more effective in abating seed damage during storage, although inclusion of neem within LDPE bags also had significant damage. Quantification of seed predating insects and diseases suggested that SuperGrainbags® also suppressed Coleopteran pests and blackspot, the latter indicative of *Fusarium graminearum*. Conversely, where farmers used LDPE bags, neem also had an additional though limited pest suppressive effect. Post-storage treatment scoring by farmers revealed a strong preference for SuperGrainbags® and no preference differences for or against neem. This study demonstrates a process by which farmers can be involved in the participatory co-design and testing of alternative wheat storage options, and stresses the need to develop SuperGrainbag® supply chains so hermetic storage can be made widely available.

## Highlights

• Surveys identified seed storage methods for participatory trial co-design in Bangladesh.
• Storage trials were then conducted with 80 wheat farming households.
• Hermetically stored seed had greater germination and coleoptile length than LDPE bags.
• Neem slightly reduced damage rates for traditionally but not hermetically stored seed.
• Both men and women farmers expressed strong preferences for hermetic storage.

## 1 Introduction

Wheat (*Triticum aestivum*) is the second most important cereal grown after rice (*Oryza* sativa) on a global basis, providing about 20% of the daily protein and calories to approximately 4.5 billion people (Shiferaw et al., 2013). Wheat is an inexpensive source of metabolizable energy and protein, generating about 20.5% of the calories and 24% of the protein consumed by 50% of South Asia's population (Shiferaw et al., 2013; FAOSTAT, 2020). Wheat is widely grown by smallholder farmers during the winter season in South Asia, though seed replacement rates are low and storage losses are considerable (Iqbal and Toufique, 2016). Losses due to improper storage are reported to be between 15 and 30% in Nepal (Devkota et al., 2018), 1.5–3.5% in Pakistan (FAO, 2013), 1.8–11.7% in India (Bala et al., 2010), and about 3.6% in Bangladesh (Bala et al., 2010; Kumar and Kalita, 2017).

In South Asia's tropical and sub-tropical climates, seed is stored during the pre-monsoon and monsoon periods from April through October. Planting of stored seed takes place six to eight months later after storage. When too much moisture is absorbed by seed during storage, its longevity and germination and hence crop establishment can be compromised (Ellis and Roberts, 1980; Vitis et al., 2020). Storage during the interim monsoon period is challenged by high relative humidity, and seeds can be predated by insects or infected by fungal diseases (Rahman et al., 1985). Common seed predators include Coleopteran weevils (Curculionidae) *Sitophilus oryzae* (L.), *Sitophilus granarius (L.),* skin beetles (Dermestidae) *Trogoderma granarium* Everts, grain beetles (Silvanidae) *Oryzaephilus surinamensis* (L.), Rust red flour beetles *Tribolium castaneum* (Herbst) and the lesser grain borer *Rhyzopertha dominica* (F.) are also common seed predators*.* These are in addition to Lepidopteran pests such as the Angoumois grain moth (Gelechiidae: *Sitotroga cerealella* (Ol.)) and some ant (Hymenoptera: Formicidae) species (Bhardwaj et al., 1977; Navarro, 2006; Kumar and Kalita, 2017). The reproduction of arthropods and hence their rates of seed predation can be exacerbated by high temperature and humidity, both of which are prevalent during the monsoon (Devkota et al., 2018). Preventing postharvest losses while maintaining seed quality is therefore crucial for farmers generally, but specifically for resource-poor farmers who may not be able to purchase fresh seed in the event of losses.

In Bangladesh, wheat is the second most widely consumed cereal after rice (BBS, 2021). It is grown on over 332,000 ha, primarily by resource-poor farmers who tend to use inexpensive, easily available and often porous materials for seed storage (Bala et al., 2010; Kumar and Kalita, 2017; Miah et al., 2018; BBS, 2021). Smallholder and resource poor farmers in Bangladesh often use available materials such as metal bins or plastic drums, and porous materials including jute bags, reused woven polypropylene fertilizer bags, or terracotta pots (Bala et al., 2010; Kumar and Kalita, 2017; Miah et al., 2018). Some farmers also add insecticides or leaves of locally available plants such as neem (*Azadirachta indica*) to stored grains (Schmutterer, 1990; Benelli et al., 2017). With insecticidal properties (Waghmare et al., 2007), neem is a fast-growing tree species native to Bangladesh, India and Pakistan (Atawodi and Atawodi, 2009). Neem leaves or seed powders have for example been effective in controlling maize weevil (*Sitophilus zeamais* Motschulsky) and bean weevils (*Callosobruchus* sp.) in Bangladesh and Ethiopia, respectively (Khatun et al., 2011; Radha, 2014; Erenso and Berhe, 2016).

Hermetically sealed containers such as those provided by GrainPro (2021) and the Purdue Improved Crop Storage bag (PICS, 2020) are increasingly promoted for use by smallholder farmers, including in Bangladesh (Baoua et al., 2013; Somavat et al., 2015; Miah et al., 2018; Baributsa and Ignacio, 2020). Hermetic storage involves sealing seed in an airtight storage container wherein an atmosphere with low O_2_ and high CO_2_ content develops through the respiration of non-seed organisms within the sealed container (Navarro, 2006; Darby and Caddik, 2007; Baributsa and Ignacio, 2020). Over time, the CO_2_ atmosphere within hermetically sealed devices smothers pests, thereby improving storage conditions and reducing losses (Navarro, 2006; Darby and Caddik, 2007; Baributsa and Ignacio, 2020). Both high density polyethylene storage bags and hermetic devices have been reported to control insect pests and maintain acceptable seed moisture levels, while being affordable to resource-poor farmers (Villers et al., 2010; Baoua et al., 2013; De Groote et al., 2013; Afzal et al., 2017; Devkota et al., 2018; Yeole and Swain, 2018). Afzal et al. (2017) also reported that maize seed stored in PICS bags had lower fungal aflatoxin contamination due to *Aspergillus* sp. than when stored in polypropylene bags.

Aside from a few studies (Somavat and Kumar, 2017; Devkota et al., 2018), there is limited assessment of the performance improved hermetic storage relative to farmers' traditional storage practices for wheat seed in South Asia. Although hermetic seed storage has been experimentally compared to other storage devices in Bangladesh, the primary focus has been on rice (Awal et al., 2017; Hossain et al., 2019a, 2019b). In many cases, studies have been laboratory-based, or when conducted under on-farm conditions, experiments have tended to be designed by researchers with apparently no to limited involvement of farmers themselves in experimental co-design or the selection of alternative storage options. In response, this study was conducted with 80 farmers in central Bangladesh, where wheat is now grown on over 41,600 ha (BBS, 2021). We employed a household survey followed by focus group discussions (FGDs) with farmers to co-design experimenmtal treatments that were managed by farmers within their own households over a 30 week storage period. Our objectives were to: (i) benchmark farmers' wheat seed storage practices as a step towards (ii) the collaborative design and implementation of farmer-participatory household wheat seed storage trials comparing the performance of hermetic SuperGrainbags® with what Miah et al. (2018) observed as farmers’ common practice of seed storage in low-density polyethylene (LDPE) bags, to additional traditional storage techniques such as neem addition, in central Bangladesh. To our knowledge, this is the largest participatory farm household trial conducted in South Asia that compares hermetic storage for wheat seed to conventional storage practices under *in situ* farm storage conditions.

## 2 Materials and methods

### 2.1 Study locations

**Fig. 1:**
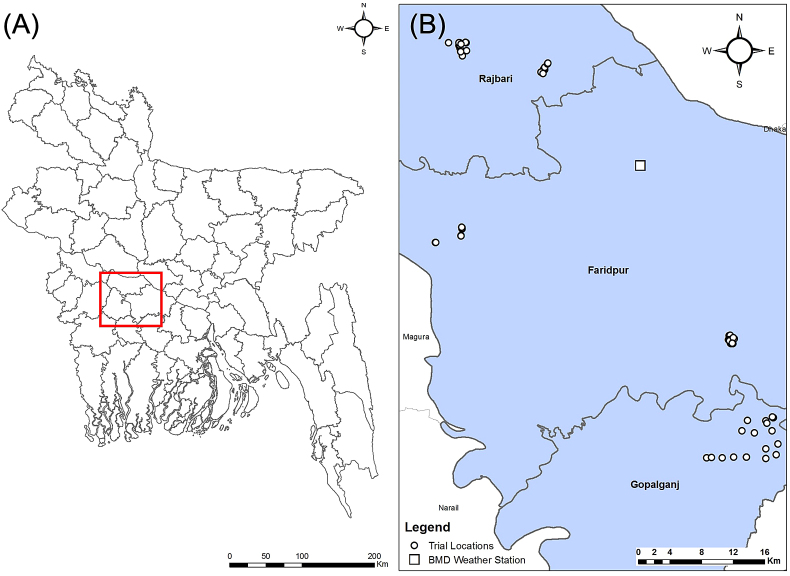
Locations of households participating in wheat seed storage trials in central Bangladesh.

Following consultations in late 2011 with the Bangladesh Agricultural Research Institute (BARI) and the Department of Agricultural Extension (DAE), studies were conducted in Faridpur, Rajbari, and Gopalgonj districts in central Bangladesh. Both BARI and DAE proposed these districts as locations in which wheat is a crop of importance to food security for smallholder farmers, but in which farm households had challenges with wheat seed storage and in which no prior experimental work evaluating wheat seed storage methods had been conducted (Fig. 1).

### 2.2 Survey of wheat farmers’ storage methods to aid in experimental design

To assist in the design of participatory household seed storage trials, we conducted a survey of 72 farm households (*n* = 28, 22, and 23 in Faridpur, Gopalgonj, and Rajbari districts, respectively) across six villages (two and three villages in Faridpur and Rajbari, respectively, and one in Gopalgonj). Villages were selected by DAE officials as locations in which field-level extension staff had an interest in research and where farmers had requested guidance on improved seed storage management recommendations. Survey respondents were first randomly drawn from a list of farm households supplied by DAE as having had at least a five-year history of growing wheat in these villages. From this initial list, DAE next identified households for survey based on criteria including (a) their willingness to participate in household trials testing alternative seed storage methods, (b) willingness to include their own saved seed in research trials, and (c) households in which male and female household heads both agreed to be surveyed. When the pre-trial surveys were administered, 72 had been the maximum number of farm households (including husbands and wives) in the selected villages that were willing to be surveyed and met the above criteria. Survey respondents thus included both male and female household heads (husbands and wives) who were interviewed separately by trained male and female enumerators, respectively, using a pretested questionnaire in February of 2012.

These pre-experiment surveys aimed to identify common wheat seed storage methods used by farmers in the study villages, while also collecting data to aid in the design of participatory household storage trials. Each interview started with a brief introduction and rapport building with farmers. This was followed by questions on what wheat varieties typically cultivated and stored, the quantity and source of seed stored, the types of storage devices typically used, use of biological or synthetic pesticides to aid storage, and the locations within the household compound where seed is stored. Additional questions on seed quality protection measures and household members responsible for seed storage were also asked.

### 2.3 Farmer's household storage trials design process and trial locations

Following initial farmer surveys, DAE staff organized the participation of husband and wife respondents together in focus group discussions (FGDs) with paired male and female discussion facilitators in each village during March of 2012. In these meetings, a descriptive summary of survey results was presented in the form of pie-charts on posters, after which a facilitated discussion led by researchers aimed to further elucidate men's and women's perceived wheat seed storage problems and interest in potential solutions. During FGDs, farmers were also introduced to the concept of hermetic storage; combined with DAE staff's partial familiarity with hermetic storage, this spurred farmers' and DAE's interest in testing hermetic SuperGrainbags® (Premium RZ), which had recently begun to be promoted for seed storage in Bangladesh. Further interactions with farmers and subsequent presentation of an initial trial protocol draft in each village in April 2012 aided in the finalization and farmers' endorsement of trial design.

### 2.4 Farmers’ household trial details and treatments

Following completion of the participatory trial design process, storage trials were initiated using the protocol co-designed with farmers in each of the villages in which farmers had been surveyed (Fig. 1). In addition to the original 72 farm households taking part in the survey, an additional eight households in the study districts that expressed interest to participate and which had grown wheat for at last five years and met on the criteria described in Section 2.2 were also included. We therefore arrived at a total of 80 participating farm households. No other selection methods or criteria were followed for these eight farmers. Trials were conducted in a completely randomized two-factor factorial design with households as replicates, with measurements for seed quality parameters taken immediately before and after seed storage.

The first experimental factor ‘neem’ had two levels - with and without neem addition - based on survey data and FGDs that highlighted how farmers sometimes mix dried neem tree leaves with stored seed to reduce pest losses. The second factor ‘storage device’ (two types: low-density polyethylene ‘LDPE’ bag and SuperGrainbags®) represented traditional and alternative storage devices, respectively. Trials were conducted under household conditions, and with the objective of assessing the performance of storage devices and methods under conditions that could be implemented by resource-poor farmers. As farmers were however largely unfamiliar with hermetic storage principles and SuperGrainbags®, all husbands and wives in each household were given a standard and brief training on how to use the experimental storage devices and maintain experimental treatments.

We supplied each farmer with a new LDPE bag, which they contained within a woven polypropylene bag, as a control treatment. Fertilizer bags in Bangladesh are typically prepared using high strength but low density woven polyethylene strips and are used to contain LDPE bags in grain storage; as such, this treatment mimicked this common farmers' practices. The second storage device was the hermetic storage Premium RZ SuperGrainbag®, hereafter reffered to as a SuperGrainbag®, provided by GrainPro Inc. (Zambales, Philippines: GrainPro, 2010; https://www.grainpro.com/grainpro-bag-zipper). The size of both the SuperGrainbags® and the LDPE bags was 70 by 40 cm, the former and later with a thickness of 78 ± 10 and 60 ± 10 μm, respectively. Each household participating in the trials therefore had a total of four bags to store seed in their houses.

All participating farmers had sufficient stock of third generation and recently harvested seed of the popular open-pollinated wheat variety Shatabdi to save in each of the treatment combinations. As not all farmers had access to the other varieties identified in pre-experiment surveys, Shatabdi was chosen by participating farmers for use in these experiments*.* Farms in Bangladesh often consist of numerous, fragmented fields and can be very small. Gautam and Ahmed (2019) for example found that cultivated land area across 62 villages in Bangladesh is declining, and averages approximately 0.32 ha. Recommended seed rates for wheat in Bangladesh are around 120 kg seed ha^−1^ (Krupnik et al., 2015). As the farmers in our study villages also cultivated other non-wheat crops during the winter, they had between 17.9 and 27.4 kg of wheat seed to store, with a mean of 25.0 ± 1.6 kg standard deviation.

Researchers commmunicated regularly with household members to assure that they did not remove any seed from the storage devices during the trial period. To achieve this goal, both male and female household heads responsible for storage were trained on the study protocol which mandated that farmers do not open storage devices during the trial period. Within each treatment, farmers stored seed that they had previously sun-dried for at least eight days of full sun on a woven date palm leaf mat in their household courtyard. Care was taken to avoid contamination and damage of seeds by insects, and all batches of seed were visually screened by farmers as being sufficiently ready for storage according to their own criteria prior to insertion into storage devices.

Following farmer's average practices as obtained from the household survey, freshly harvested neem leaves were collected from trees between three and eight years of age located in the vicinity of each farmer's household. These leaves were air dried under shade provided by trees in farmers' household compounds over a period of at least eight days to a constant weight. Dried neem leaves were then crushed by hand for 1 minute. Crushed dried leaves equivalent to 4% weight of each quantity of seed stored (between 0.71 and 1.09 kg, with an average of 1 kg) were subsequently mixed with seed before storage. The quantity of neem applied was chosen by households during FGDs as an average of what they considered to be the requisite ratio for neem leaves to seed stored (see Section 3.1). As with wheat seed, care was taken to avoid the contamination of neem with foreign organisms, and only neem that farmers judged to be of sufficient quality was mixed with seed. Wheat seeds and/or neem leaves were then placed within a SuperGrainbag*®* or LDPE bag. Farmers also employed their own used woven propylene bags to enclose the SuperGrainbags® and LDPE bags. The propylene bags also provided support and reduced risk of inner-bag damage during storage.

After filling both LDPE bags with seeds and/or neem, excess air was removed by squeezing the bags from the bottom up until no more air could be vacated. The tops of the bags were then twisted to close them. This reduced the potential for introduction of additional air within the bags. This also mimicked farmers’ own practices for preparing storage for LDPE bags, which were then tightly closed and sealed with jute twine. SuperGrainbags® were conversely prepared using identical practices and in accordance with management guidelines advised by GrainPro (2010), though rather than being twisted and closed with twine, they were sealed with a specialized ziplock and fastener provided with the bag. Enclosing fertilizer bags were then similarly sealed for both SuperGrainbags® and LDPE bags with jute twine.

Storage devices were next then placed on a single “*macha”,* a type of table at least 0.6 meters in height made of wood or bamboo, which were located inside each participant's household in a location of their choice. Storage devices were arranged in completely randomized order on the *macha* in each household, with at least 30 cm between devices and care taken by farmers to not place any other objects on top of the bags or the *macha*. To further isolate wheat seed and avoid any cross-contamination risks, no seeds or grain of other crops were stored within the same room in farmers' households. Similarly, no farmers stored seed in their kitchens or other locations within the household that could increase risk of abnormally high humidity, seed predation, or contamination events.

**Fig. 2:**
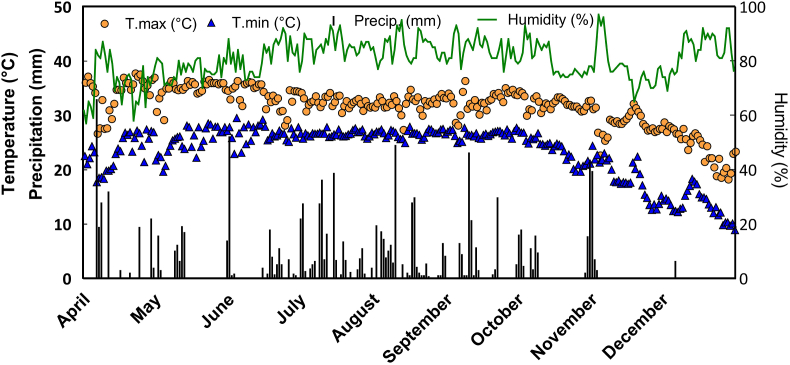
Observed weather patterns during the wheat storage trial period from April–November 2013 measured at the Faridpur observatory of the Bangladesh Meteorological Department (23°35′56.09"N, 89°50′44.17"E). T. max and T. min indicate maximum and minimum daily temperature, respectively. Precip. refers to precipitation.

Depending on the household, storage trials began between April 25 and May 8 and were concluded between November 10 and 25 in 2012, when farmers deemed it time to retrieve seed for planting. Locations of the participating farmer households are presented in Fig. 1. Fig. 2 provides weather data during the storage trials period collected from Bangladesh Meteorological Department weather station in Faridpur (23°35′56.09"N, 89°50′44.17"E).

### 2.5 Data collection

#### 2.5.1 Pre- and post-storage seed quality assessment

Grain moisture content was measured using a calibrated grain moisture meter (G-Won HiTech; model GMK303RS, Seoul, Korea) both immediately before and after storage. Following recommendations from BARI for on-farm experiments with a large number of replicates and treatments, we retrieved five randomly selected sub-samples of 50 seeds both before and after storage. Seed germination testing was conducted immediately thereafter in BARI's Seed Testing Laboratory in Gazipur. The five sub-samples of 50 seeds from each treatment combination (a total of 250 seeds) from each household were first visually examined for signs of fungal presence and damage signified by cracking or evidence of predation. They were then placed evenly on a moistened and a distilled water damped germination paper towel at 8 a.m. and placed in an incubator at 20 °C for eight days. Moisture was maintained by misting with distilled water at 8 a.m., 2 p.m. and 8 p.m. daily. After the eighth day, normally germinated seeds that showed emerged sprouts were distinguished from dead and abnormally germinated seeds and subsequently counted from each of the five sub-sample groups. Sub-sampled data were then averaged to provide a single germination percentage for each treatment from each household replicate. In addition to germination, seed vigor can be a useful parameter for identifying the potential of seed to perform well after storage (Marcos Filho, 2015). Hence, we also measured the coleoptile length of all germinated seeds within each of the five sub-samples from each treatment combination and data averaged to provide a single coleoptile length for each treatment from each household replicate.

#### 2.5.2 Post-storage pest assessment

Prior to removing seed from storage devices after storage, four composite samples of seed equal to 500 g were randomly retrieved from storage devices and placed in bowls. Samples were then thoroughly examined following Adams and Schulten (1978) to identify and quantify insects from nymphal stage to adult in each of the four samples by taxonomic order and family. We also retrieved two sub-samples of 50 randomly selected seeds from each 500 g batch, for a total of 400 seeds per treatment per replicate, and counted the number of seeds that had black or brown spots indicative of disease (Rinjani et al., 2019). Data were then averaged among sub-samples and extrapolated for each treatment and replication in proportion to the weight of each stored batch of seed.

#### 2.5.3 Farmers’ performance assessment

At the conclusion of storage, male and female household heads together extracted seed from storage devices. Following a visual inspection, they were each independently requested to provide a score between 1 and 4 representing their perception of the effectiveness of each treatment option to maintain seed quality, with 1 being poor, and 4 being high, performance.

### 2.6 Statistical analysis

Farm household survey data across villages and districts were analyzed descriptively and presented as percent total in pie-chart form to farmers during FGDs, and in tabular form in this manuscript. Data for different parameters observed in the household storage trials were analyzed using three models. In the first statistical model, before and after storage measurements of seed moisture content, germination, coleoptile length and damaged seed were analyzed using a repeated measures two-factor factorial ANOVA, with the addition or absence of neem as the first within-subjects factor, and storage device type as the second within-subjects factor. The timing of sampling (before or after storage) was considered as the between-subjects factor. Both within- and between-subjects factors were treated as fixed effects, with replicate considered as a random effect. This model permits the examination of two- and three-way interactions that compare the second (with or without neem) and third (SuperGrainbag® or LDPE bag) factor treatment performance to each other, while accounting for any variation observed in the first factor (before and after storage). In the second statistical model, post-storage data for insects and indicators of disease were analyzed using a factorial ANOVA, with neem (with or without), and storage device (LDPE or hermetic), as the first and second factors, respectively. These factors were also considered as fixed, while farmer replicate was again treated as a random effect. Finally, in the third statistical model, farmer's post-storage preference scoring for storage methods was analyzed using a three-factor design/ANOVA, with storage device, neem, and sex of the survey respondents considered as fixed factors, with household replicates as a random factor.

All analyses were conducted using the restricted maximum likelihood (REML) procedure in JMP (Version 14; SAS institute Cary, NC, USA; Littell et al., 2006). Data that were not normally distributed were subject to logarithmic transformation (log[*X* + 1]), where *X* is the original observation) before they were subjected to ANOVA. If significance was detected at α = 0.05, treatment means were separated by using the Student's-*t* or Tukey's Honestly Significantly Different (HSD) test for non-interacting and interacting sources of variation, respectively.

## 3 Results

### 3.1 Farmer pre-experiment survey to inform research design

Initial surveys conducted prior to the start of the trials (see section 3.2) indicated that farmers in the study villages cultivated a diversity of wheat varieties including Shatabdi, Prodip, Bijoy, Kanchan, and BARI Gom-26 using second to fourth generation seed. Eighty percent of the respondents in these villages however grew the first two varieties, with much fewer cultivating the remainder. Most of respondents reported that primarily female household members were responsible for drying and conserving wheat seed over the monsoon, while both men and women (12% of the samples) showed joint responsibility to achieve this goal. Women were also primarily responsible for assuring seed germination (48.3% of the cases). Approximately 80% of respondents indicated that they saved and used their own seed in the next season after thorough sun drying (Table 1). Farmers typically saved seed for between three to five generations before repurchasing new seed.

**Table 1:** Farmers' responses regarding stored grain pests, and storage devices and methods used for wheat storage obtained through a survey prior to start of the storage trials in 2012, in central Bangladesh (*n* = 72).

Parameter	%	Parameter	%
Storage devices used by farmers		Surface for keeping seed storage devices	
Jute sack	13.3	*Macha*^b^	56.2
Reused fertilizer bag	4.1	Wooden surface	30.1
LDPE bag alone^a^	16.3	Brick	8.2
LDPE with reused fertilizer bag	42.9	Other (Chair, bag, Tire, Floor)	5.5
Metal or plastic drum	17.3		
Terracotta pot	4.1		
Others (Dole, cement pot)	2.0		
	
Farmers' perceived causes of storage losses		Storage protection measures employed by farmers	
Rodents	9.4	Various insecticides	10.0
Insect pests	28.1	Neem leaves mixed with seed	14.3
Moisture	54.7	Naphthalene	12.9
Other	7.8	Rodenticides	1.4
		No protection measures used	61.4
	
Farmers' seed drying methods before storage		Substrates used for germination testing by farmers	
Ground	28.6	Coconut Husk	31.4
Polythene surface	14.3	Banana stem sheath	35.7
Cloth surface	5.2	Wet soil	25.7
Katha surface	1.3	Wet jute sack	7.2
Jute sack surface	9.1		
Woven date palm leaf mat	33.8		
Others (Plastic bag, Tripoli)	7.7		
	
Main responsibility during storage		Main responsibility for checking seed germination	
Adult men	1.5	Adult men	25.9
Adult women	86.8	Adult women	48.3
Both women and women	11.7	Both women and women	25.8
	
Seed grading devices used by farmers		Farmer's experience with formal training on storage	
Winnow	67.6	Yes	28.6
Bamboo sieve	32.4	No	71.4

a Low density polyethylene (LDPE) bag.

b A raised platform with a surface made of bamboo.

Prior to storage, 34% of the respondents indicated that they typically sun-dried seeds on a woven date palm leaf mat, while 27% dried seed by spreading it directly on the ground within their household compounds. Another 14% sun dried seed on a polyethylene surface, while 9% spread and dried on jute sacks. The time required for wheat seed drying ranged from 4 to 12 days across surveyed households. Surveyed respondents indicated that they determined the appropriate level of seed dryness before commencing storage by biting into their seed, which were considered to be sufficiently dried when a ‘metallic’ and crunching sound was produced during chewing. No households were observed to use electronic meters or other equipment to judge moisture levels for drying before storage.

Seed was typically stored for 30–32 weeks in different types of storage devices. Forty-three percent of households surveyed stored wheat seed in LDPE bags inserted inside a reused fertilizer bag, 17.3% metal or plastic drums, 16.3% a LDPE bag alone, and the remainder used other devices. No farmers had any previous experience with hermetic storage. Fifty-six percent of the respondents placed their storage devices on a '*macha'*, 30% on a wooden surface, 8% on bricks, and remaining on other surfaces.

During FGDs, farmers expressed that neither they could neither reliably access nor afford synthetic insecticides as an additive to stored wheat seed. For this reason, they expressed an interest in experimenting with neem leaves, which are occasionally used in the study area (Table 1) as a potential alternative to reduce pest attack of stored seed. Farmers perceived moisture as the main cause of damage to wheat seed during storage, followed by diseases and insects. Yet, while 61% of the respondents indicated that they did not take any additional protection measures against insect damage or diseases, 14% suggested they commonly used dried neem leaves at an average rate of 4% weight relative to the quantity of seed stored. An additional 13% of households used 1–2 naphthalin (C_10_H_8_) tablets per storage bag, although most indicated that naphthalin was expensive and regularly not available in the market. The remaining households used various types of insecticides (though farmers were usually unable to recall active ingredients or even commercial brand names) as protection measures. Only 1% of farmers surveyed used rodenticides.

### 3.2 Seed storage household trials

As described in Section 2.3, the results from pre-trial surveys were presented to each household participating in this study during FGDs conducted in each village. Pie-charts depicting the percent of farmers using different seed drying and storage techniques were prepared on posters and shown to farmers and extension agents to facilitate discussion and inform the design of participatory household seed storage trials.

#### 3.2.1 Seed moisture content

**Fig. 3:**
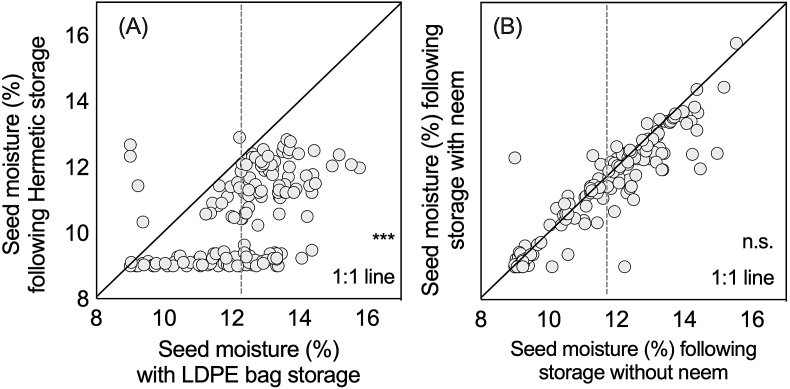
1:1 plots for post-storage seed moisture (%) considering the factors (A) hermetic SuperGrainbag® performance to LDPE bag performance, and (B) seed storage both with and without the addition of dried neem (*Azadirachta indica*) leaves in household wheat storage trials in 2012 in central Bangladesh. The vertical dotted line indicates the 12.5% moisture threshold for seed storage recommended by Villers et al. (2010) (*n* = 80).

Prior to storage, the mean seed moisture across households was 8.53%, with no significant differences across treatments (Table 2). However, following storage, ANOVA results showed a significant (*P* < 0.001) mean increase (2.8%) in moisture across treatments. The inclusion of neem had no effect on seed moisture, though a significant (*P* < 0.001) three-way interaction was observed, with SuperGrainbags® preventing seed moisture from rising above 10.4%, while seed moisture in LDPE bags exceeded 12.1%. Seed moisture content after storage demonstrated that of the 160 first factor treatment comparisons, 103 of the observations of LDPE storage had moisture contents >12% before storage, while SuperGrainbags® had only 28 (Fig. 3A). Conversely, fewer differences were observed when comparing neem addition to no neem between storage device types (Fig. 3B).

**Table 2:** Seed quality parameters, and blackspots and insect infestation (±SEM) measured before and after storage in wheat seed storage trials among farmers in 2012 in central Bangladesh (*n* = 80).

Variation source	Seed quality parameters			
	Seed moisture content (%)	Germination (%)	Coleoptile length (mm)	Damaged seed (%)
Sampling time (ST)				
Before storage	8.53 (0.79) b	97.69 (0.08) a	58.12 (1.26) a	0.49 (0.02) b
After storage	11.34 (0.93) a	95.45 (0.37) b	53.83 (0.78) b	2.03 (0.13) a
Neem (N)				
With Neem	9.89 (1.14)	96.70 (0.26)	54.91 (1.02)	1.04 (0.08) b
Without Neem	9.98 (1.21)	96.45 (0.30)	57.04 (1.08)	1.48 (0.12) a
Storage device (S)				
SuperGrainbag® (SGB)^a^	9.46 (0.95) b	97.52 (0.10) a	56.34 (1.02)	0.84 (0.05) a
LDPE^b^	10.41 (1.31) a	95.63 (0.37) b	55.61 (1.08)	1.68 (0.13) a
Interactions				
ST × N				
Before storage, with neem	8.55 (1.17)	97.63 (0.11) a	56.24 (1.72)	0.52 (0.22) c
Before storage, without neem	8.52 (1.07)	97.76 (0.10) a	59.99 (1.82)	0.47 (0.22) c
After storage, with neem	11.23 (1.25)	95.77 (0.49) b	53.57 (1.08)	1.57 (0.15) b
After storage, without neem	11.45 (1.38)	95.15 (0.56) b	54.10 (1.11)	2.49 (0.22) a
ST × S				
Before storage, with SGB	8.51 (1.19) c	97.73 (0.10) a	55.89 (1.72) bc	0.48 (0.22) c
Before storage, without SGB	8.55 (1.05) c	97.67 (0.11) a	60.34 (1.82) a	0.50 (0.22) c
After storage, with SGB	10.40 (1.04) b	97.30 (0.17) a	56.79 (1.12) ab	1.19 (0.10) b
After storage, without SGB	12.28 (1.14) a	93.61 (0.70) b	50.88 (1.03) c	2.87 (0.22) a
N × S				
With N and with SGB	9.41 (1.40)	97.44 (0.16)	54.40 (1.41)	0.74 (0.06) c
Without N but with SGB	9.51 (1.29)	97.60 (0.13)	58.28 (1.47)	0.98 (0.09) bc
With N and with LDPE	10.37 (1.72)	95.96 (0.48)	55.41 (1.47)	1.35 (0.14) b
Without N but without LDPE	10.46 (1.95)	95.31 (0.56)	55.81 (1.58)	2.02 (0.22) a
ST × N × S				
Before storage: With N and SGB	8.42 (1.80) c	97.74 (0.15)	52.60 (2.341)	0.53 (0.03) d
Before storage: Without N but with SGB	8.60 (1.55) c	97.73 (0.14)	59.18 (2.48)	0.48 (0.03) d
Before storage: With N and LDPE	7.67 (1.49) c	97.53 (0.16)	59.88 (2.48)	0.51 (0.03) d
Before storage: Without N but with LDPE	8.43 (1.48) c	97.80 (0.15)	60.80 (2.69)	0.49 (0.03) d
After storage: With N and SGB	10.40 (1.48) b	97.14 (0.27)	56.20 (1.58)	0.95 (0.12) cd
After storage: Without N but with SGB	10.41 (1.48) b	97.47 (0.21)	57.37 (1.58)	1.42 (0.16) c
After storage: With N and LDPE	12.07 (1.53) a	94.39 (0.93)	50.94 (1.43)	2.19 (0.25) b
After storage: Without N but with LDPE	12.49 (1.65) a	92.82 (1.04)	50.82 (1.48)	3.55 (0.36) a
*F*-Values				
ST	362.38***	28.88***	4.38*	99.07***
N	1.12^NS^	0.67 ^NS^	2.79 ^NS^	16.96***
S	74.99***	21.01***	0.37 ^NS^	48.62***
ST × N	1.96 ^NS^	1.56 ^NS^	1.59 ^NS^	20.75***
ST × S	68.79***	19.32***	18.47***	47.11***
N × S	0.00 ^NS^	1.69 ^NS^	2.31 ^NS^	6.20*
ST × N × S	6.26**	3.02^φ^	0.91 ^NS^	4.59*

Letters in columns not separated by row breaks indicate significant differences at α = 0.05 according to Tukey's HSD test. Total df for this repeated measures model is 1,158 for all variation sources.. ***, ** and * indicates significances at *P* < 0.001, 0.01 and 0.05, respectively; NS indicates non-significance. ^φ^ indicates nearly significant at *P* = 0.08.

a GrainPro Zipper SuperGrainbags®.

b LDPE indicates low density polyethylene.

#### 3.2.2 Germination percentage, coleoptile length, and damaged seed

Both germination percentage and coleoptile length significantly (*P* < 0.001 and *P* < 0.05, respectively) decreased across treatments following storage. The percentage of damaged seed conversely significantly (*P* < 0.001) increased by 1.5% across treatments (Table 2). While inclusion of neem had no effect on germination before and after storage, a significant (*P* < 0.001) interaction between sampling time and storage device was observed, with SuperGrainbags® maintaining a germination percentage when compared to pre-storage levels. LDPE bags however were associated with significantly reduced germination by 3.7–4.1% lower than pre-storage levels. A nearly significant (*P* = 0.08) three-way interaction was also observed, with SuperGrainbags® maintaining germination percentage on-par with pre-storage conditions, yet with neem inclusion to LDPE bags tending towards a slight increase. As with germination percentage, significant (*P* < 0.001) differences between storage devices were found for coleoptile length when comparing pre- and post-storage conditions. Coleoptile lengths measured following SuperGrainbag® storage were not different than pre-storage lengths. Conversely, coleoptile lengths were 5.0–9.5 mm shorter, on average, following storage in LDPE bags, and were significantly different from pre- or post-storage SuperGrainbag® measurements.

**Fig. 4:**
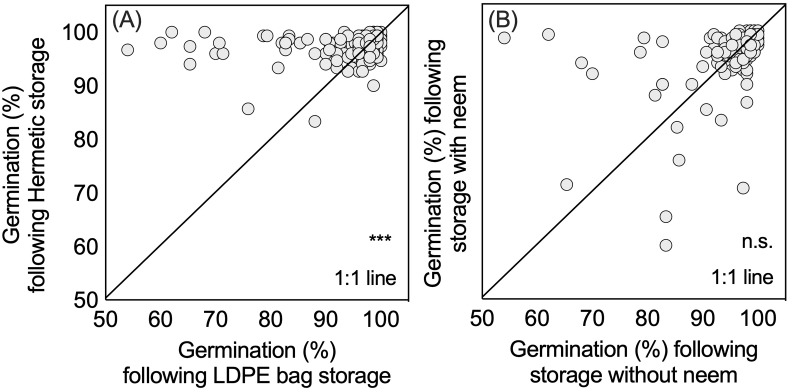
1:1 plots for post-storage seed germination rates after eight days (%) considering the factors (A) hermetic SuperGrainbag® performance to LDPE bag performance, and (B) seed storage with and without the addition of dried neem (*Azadirachta indica*) leaves in household wheat storage trials in 2012 in central Bangladesh (*n* = 80).

**Fig. 5:**
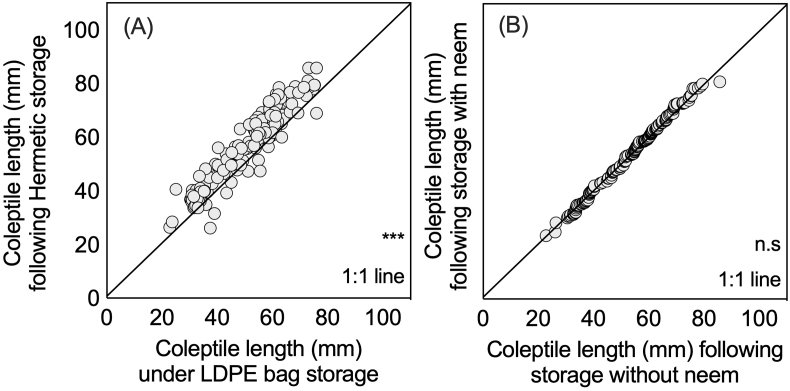
1:1 plots for post-storage seedling coleoptile length (mm) considering the factors (A) hermetic SuperGrain Bag performance to LDPE bag performance, and (B) seed storage with and without the addition of dried neem (*Azadirachta indica*) leaves in household wheat storage trials in 2012 in central Bangladesh (*n* = 80).

Data points for each of the individual measurements of germination percentage and storage device after storage revealed 89 measurements with hermetic resulting in greater germination compared to 71 with LDPE storage (Fig. 4A and B). Likewise, 89 observations with neem had greater germination compared to 71 without neem. Similarly, for 149 out of 160 measurements, SuperGrainbags® had greater coleoptile length than the LDPE bags (Fig. 5A and B).

There was a significant interaction (*P* < 0.001) between sampling time and inclusion of neem. Each two- and three-way interaction that considered sampling time (before or after storage) as a factor affecting seed damage was significant at either *P* < 0.05 or *P* < 0.001. While no differences between treatments were found, and damage levels were low (0.3–0.5%) before storage, the addition of neem to SuperGrainbags® maintained these low levels, while SuperGrainbags® without neem had a significantly higher damage rate of 1.4% after storage. Considering the LDPE bag-based treatments, use of neem lowered damage rates from 3.6 to 2.2% compared to LDPE bags alone, though both LDPE treatments (with or without neem) had higher damage than the SuperGrainbag® treatments.

#### 3.2.3 Insect and disease pests

Formicidae, Acaridae and Lepidopteran arthropods were all observed during post-storage sampling, although the latter two were found at extremely small population densities (< 2 individuals each in the entire study) and no differences among treatments. Our household surveys also suggested that insect pest presence was dominated by Coleoptera. The primary family identified was Curculionidae, with species including *S. oryzae* and *S. zeamais.* Aside from beetles found in the weevil family, we also identified individuals from the Chrysomelidae, Tenebrionidae and Bostrichidae families. As the populations of these families were variable and small in number, we grouped them into one functional group, and compared them to the density of Curculionidae as they were relatively more abundant. Insect density (individuals kg^−1^ stored seed) ranged from 0 to 6 for the former family and from 0 to 9 for the groupings of Chrysomelid, Tenebrionid and Bostrichid beetles, with a mean density of 0.5 for Curculionidae and the grouping of other beetles (Table 3).

**Table 3:** Insect and blackspot infestation (±SEM) measured after storage in wheat seed storage trials among farmers in 2012 in Bangladesh (*n* = 80).

Variation source	Pests and diseases observed following storage		
	Coleoptera population density (individuals kg seed^−1^) by family		Blackspot (%)^e^
	Curculionidae^c^	Other^c^^,^^d^	
Neem (N)			
With Neem	0.32 (0.05) b	0.26 (0.06) b	3.06 (0.16) a
Without Neem	0.57 (0.07) a	0.61 (0.12) a	3.97 (0.21) b
Storage device (S)			
SuperGrainbag® (SGB)^a^	0.15 (0.02) b	0.03 (0.0) b	2.70 (0.16) b
LDPE^b^	0.74 (0.08) a	0.84 (0.12) a	4.33 (0.20) a
Interactions			
With N and SGB	0.11 (0.03) c	0.00 (0.00) c	2.55 (0.24) c
Without N but with SGB	0.19 (0.04) c	0.07 (0.0) c	2.85 (0.22) bc
With N and LDPE^b^	0.54 (0.10) b	0.54 (0.12) b	3.57 (0.21) b
Without N but with LDPE	0.95 (0.13) a	1.14 (0.21) a	5.09 (0.33) a
*F*-Values			
N	4.0*	8.4**	19.6***
S	48.6***	46.2***	52.35***
N × S	8.5**	4.8*	8.0*

Letters in columns not separated by row breaks indicate significant differences at alpha = 0.05 according to Tukey's HSD test α = 0.05. ***, ** and * indicates significances at *P* < 0.001, 0.01 and 0.05, respectively; NS indicates non-significance. df for the model is 3, with an error of 216 and total of 219 for all variation sources.

a GrainPro Zipper SuperGrainbags®.

b LDPE indicates low density polyethylene.

c Data were log+1 transformed prior to ANOVA. Back-transformed data are shown here.

d Including Chrysomelidae, Tenebrionidae and Bostrichidae. Formicidae, Acaridae, and Lepidopteran arthropods were also observed but at extremely small densities and no differences among treatments. They are therefore not shown.

e Blackspot can result from attacks by *Fusarium graminearum* (Rinjani et al., 2019).

Significant differences (*P* < 0.05) were found for populations comparing with and without neem addition (Table 3), with higher numbers of beetles for the latter (0.57 individuals kg^−1^ seed stored) than the former (0.32 individuals). Similarly, hermetic storage without neem had a highly significant effect (*P* < 0.001) on reducing weevil density, with 0.15 individuals kg^−1^ seed stored compared to when LDPE storage without neem (0.74 individuals). A significant (*P* < 0.01) interaction was also found when comparing both factors. In a statistical sense, hermetic storage with or without neem were both equally suppressive of Curculionidae populations, although for LDPE storage, neem addition lowered weevil density by 0.41 individuals kg^−1^ seed stored (Table 2).

Considering non-Curculionidae species, Chrysomelid beetles such as bean weevil (*Callosobruchus* sp.), Tenebrionid beetles such as red flour beetle (*Tribolium castaneum*) and darkling beetle (*Coleoptera tenebrionidae*), and Bostrichid beetles such as auger beetles (*Heterobostrychus* sp.) and borers beetles (*Rhyzopertha dominica*) were found. A single lined flat bark beetle *Laemophloeus minutus* of the superfamily Cucujoidea was also observed. As each of the population density of these species was relatively low on an individual basis, we bulked all non-Curculionidae beetles into a general category for analysis. Neem addition had similar effects as found with Curculionidae with significant (*P* < 0.01) differences, and population density 0.35 individuals kg^−1^ seed stored lower than without neem. Hermetic storage had a similar suppressive effect on population density of Coleoptera, with highly (*P* < 0.001) significant differences between LDPE bags and SuperGrainbags®, the latter nearly eliminating non-weevil beetle populations relative to the former. A mildly significant (*P* < 0.05) interaction was also found when these factors were compared, mirroring the same pattern as found for Curculionidae.

Following storage, we observed blackspots on seeds indicative of attack by fungus of Order Hypocreales family Nectriaceae *Fusarium graminearum*. Blackspotted seeds ranged from 0 to 15% across all observations but were lowest under hermetic storage either with or without neem. The percentage of seeds with blackspots was significantly (*P* < 0.001) different when neem was added, though at low levels of one percentage point less when neem was added to storage devices. SuperGrainbags® had a slightly stronger and significant (*P* < 0.001) effect, with an incidence of blackspot 1.6% less than LDPE bags. Mildly significant (*P* < 0.05) interactions were observed, with the highest percentage of blackspot (5.1%) in LDPE bags without neem and dropping to 3.5% in LDPE bags with neem, the latter statistically similar to the degree of disease suppression observed with SuperGrainbags® without neem. Neem addition to SuperGrainbags® however had the greatest (2.9%) blackspot suppressive effect.

#### 3.2.4 Farmers’ preferences and feasibility in Bangladesh

After storing seeds in the LDPE bags and SuperGrainbags® for 30 weeks with or without neem, both male and female (husband and wife) household members indicated significantly greater (*P* < 00.01) preference for SuperGrainbags® compared to LDPE bags for wheat seed storage (data not shown). No differences in preferences were found between men or women, nor for inclusion or exclusion of neem, under any storage device option.

## 4 Discussion

### 4.1 Pre-experiment farmer survey on wheat storage methods and issues

The results of the pre-experiment household survey in the study villages showed that female household members in central Bangladesh were primarily responsible for drying and conserving wheat seed over the monsoon season. These findings were consistent with previous observations for northern Bangladesh (Meisner et al., 2003). Surveyed respondents also indicated that they determined the appropriate level of seed dryness before commencing storage by biting into their seed. These observations were also similar for farmers who store rice seed (Hossain et al., 2016; Oakley and Momsen, 2007) or wheat seed (Meisner et al., 2003) in northern Bangladesh. Although none of the households used electronic meters or other equipment to judge moisture levels for drying before storage, Hossain et al. (2016) encouraged to use such practices to improve wheat seed storage in Bangladesh.

Surveyed farmers in study villages perceived moisture as the main cause of damage to wheat seed during storage - an observation consistent to that for rice in northern Bangladesh (Hossain et al., 2016). The current study also showed that only about 39% farmers used various measures including dried neem leaves, naphthalin tablets, and various types of insecticides and rodenticides. This observation on use of dried neem leaves confirms reports by Benelli et al. (2017) and Schmutterer (1990) that farmers may perceive neem application to seed storage containers as beneficial for maintaining seed quality.

### 4.2 Seed storage household trials

#### 4.2.1 Seed moisture content

Prior to storage, there were no significant differences in mean seed moisture content across treatments (Table 2). Together with such non-significant differences and though care was taken to fully close each storage device tightly, there was some increase in seed moisture content after storage. Increased moisture could also potentially be attributed to non-seed organisms within storage containers (see section 3.2.3 for details). Previous researchers also reported that there is always some risk of exogenous moisture entering storage devices and contributing to increase in seed moisture content (Afzal et al., 2017; Baributsa and Ignacio, 2020; Devkota et al., 2018). Villers et al. (2010) suggested that when wheat seed is stored at less than 12.5% moisture content and is free of insects or disease-causing organisms, moisture in the upper layers of the grain will increase insignificantly. On contrary, when it is stored at above this level, the high moisture carried into the upper layers of the seed will risk the formation of mold (DPI, 2019).

#### 4.2.2 Germination percentage, coleoptile length, and damaged seed

The current study showed that wheat stored in SuperGrainbags® and with neem addition had greater coleoptile length and higher germination percentage than that stored in the LDPE bags and without neem. The coleoptile is the sheath that protects the emerging gramineous species shoot tips that penetrate up and through the soil. The speed at which it emerges from the soil is an important indicator of a seedling's ability to establish itself in the field and contribute to adequate crop establishment after seeding. Finch-Savage and Bassel (2016) and Rebetzke et al. (2019) also reported that rapid seedling establishment is a desirable trait for the crop's successive growth and development, including in wheat. However, to our knowledge, similar observations of coleoptile expansion have not previously been reported in the literature; as such, they should be treated with caution, with confirmatory and exploratory research needed to validate and identify the physical or biological basis for observed effects. Similarly, future research should include additional measures of seedling vigor that we were not able to quantify in our study, including shoot and root dry weights, and root length.

The current study also suggests that neem can ameliorate wheat seed damage during storage. The addition of neem to wheat seed in SuperGrainbags® maintained low damage levels, while seed without neem in SuperGrainbags® had a slightly but significantly higher damage rate after storage. The LDPE bags with neem had lower damage rates compared to LDPE bags alone, though both LDPE bags with or without neem had higher damage than the SuperGrainbags®. Al-Yahya (2001) reported that physical damage to seed can affect wheat germination. Morrison (1999) stressed that such damage to seed becomes an important parameter in seed health testing for lot assessments.

The characteristics of high germination rate and rapid coleoptile expansion is desirable where farmers sow their wheat seed deeply into the soil, or in zero or reduced tillage systems where shoot emergence may be hampered (Morris et al., 2010). Our observations suggest that both seed vigor parameters can be improved with the use of hermetic storage. Given the emphasis in many agricultural research and development programs on resource-conserving practices such as zero tillage in South Asia and in Bangladesh (Dixon et al., 2020; Gathala et al., 2021), further research should consider examining the potential for hermetic storage to improve wheat establishment under zero or reduced tillage under field conditions. Lastly, our germination testing methods are a local adaptation of those proposed by International Seed Testing Association and the Association of Official Seed Analysts, and were advised by BARI given the nature of our on-farm research and collection of a large number (*n* = 80) of replicate samples. The ISTA (2022) and AOSA Rules (2011) and germination methods were developed primarily to evaluate commercially produced and large volume seed lots for international trade with a lower number of replicate samples than collected in this study. However, future research could compare the methods applied in the current study to those of the ISTA and AOSA.

#### 4.2.3 Insect and disease pests

The results of the current study suggest that both neem addition and/or hermetic storage could be used to reduce densities of Coleopteran pests when storing wheat over the monsoon in the sub-tropics. During storage, insect and disease-causing organisms’ activities release moisture and energy in the form of heat into the spaces between seeds. Moisture therefore builds up faster and to higher levels from insects and other organisms than from grain respiration alone (Reed et al., 2007). Our observations of seed predating arthropods following storage may therefore help explain some of the difference in seed moisture content discussed in Section 3.2.1. Significant interactions for both Curculionidae and the groupings of other beetles also suggest that neem addition at rates commonly used by farmers (approximately 4% dried neem leaves to seed by weight) can potentially suppress grain predating beetle pests, although use of hermetic storage bags – either with or without neem – suppressed populations nearly completely. Further research however should evaluate different rates of neem addition to these and other storage options to evaluate if improved pest suppression can be achieved.

Insects can also enhance fungal development because they increase moisture and temperature, open the testa for infection, and can supply inoculum. The fungal pathogen *Fusarium graminearum* can cause blackspots and molds can cause mycotoxins and aflatoxins in stored grain (Alshamaq and Yu, 2017; Bennett and Klich, 2003). Our results showed that blackspotted seeds were lower under hermetic storage than LDPE storage either with or without neem addition and were much lower when neem addition was considered. SuperGrainbags® had a slightly stronger and significant (*P* < 0.001) effect, with lower incidence of blackspot than LDPE bags. Mildly significant (*P* < 0.05) interactions were observed, with the higher percentage of blackspotted seed found for LDPE bags without neem than for LDPE bags with neem, the latter statistically similar to the degree of disease suppression observed with SuperGrainbags® without neem. Neem addition to SuperGrainbags® however had the greatest blackspot suppressive effect.

The presence of mycotoxins and aflatoxins as reflected by the presence of molds and blackspots in stored grain suggests the need for the development of control measures to avoid potential losses due to molds and fungal and insect attacks (Alshamaq and Yu, 2017; Afzal et al., 2017; Bennett and Klich, 2003). An important development in the improvement of grain quality and control of storage diseases is the chemical determination of mycotoxins. The presence of toxic metabolites may reflect the deterioration of grain and indicate possible feed and food hazards. In this study, although blackspots in the seed samples were generally low, they are likely to be representative of pathogens and hence need to be treated with appropriate care. Knowledge on the extent of mold-induced mycotoxins and resulting wheat seed losses to aflatoxin during storage in smallholder farming households in South Asia and Bangladesh is lacking. In more developed countries, inhibition of disease infection and growth during storage is usually accomplished by modification of the inter-seed environment (i.e., moisture, temperature, and atmosphere) in industrial-scale storage facilities (Tuite and Foster, 1979). Such facilities are complex and capital intensive; and as such, under smallholder farming circumstances, the storage options described in this study are likely to be among a range of more appropriate options for adoption. Although we were able to provide general information on weather conditions in the study area (Fig. 2), future research should measure micro-climatic conditions within storage rooms within farmers’ households to provide further inference on the environmental conditions that may affect storage device performance. Moisture, temperature and atmosphere within storage devices could also be considered. Lastly, it is important to note that recent studies have begun to emerge that indicate increased tolerance of some insect species to lhypoxic environments through modification of their aerobic metabolism different lifecycle stages (Cao et al., 2019). Although we found near complete suppression of Coleopteran pests with hermetic storage, further research should therefore seek to provide detailed characterization of survival and the relative abundance at a species level.

#### 4.2.4 Farmers’ preferences and feasibility in Bangladesh

At the conclusion of the storage period, when participating farmers were asked about the relative preference of different storage treatments both male and female (husband and wife) household members indicated significantly greater (*P* < 00.01) preference for SuperGrainbags® compared to LDPE bags for wheat seed storage. There were however no differences in preferences between men or women farmers, nor for inclusion or exclusion of neem, under any storage device option. These data suggest that farmers consider SuperGrainbags® to be an effective device for wheat seed storage and that the co-design and participatory implementation of trials by farming households can help inform opinions with experience and evidence. Farmers however also expressed concerns about the SuperGrainbag® as they are not produced domestically and are still not widely or commercially available in Bangladesh. The current cost for an imported SuperGrainbag® is around USD 1.9 compared to USD 0.4 for a locally made LDPE bag. Although these costs appear to be miniscule, farmers who have not participated in trials may have an aversion to investing in hermetic storage out of a perception that LDPE bags are sufficient, especially when used in combination with neem and/or insecticides. Such costs are also higher than those reported by Devkota et al. (2018), who noted little difference between SuperGrainbags® and farmers’ traditional storage methods in Nepal.

We suggest that further action research be conducted by extension services to familiarize farmers with the viability of different storage options. Research to identify Bangladeshi farmers’ willingness to pay for SuperGrainbags® could also help to inform commercialization efforts and/or assist in the development of supportive market policy and incentives for farmers to purchase and access hermetic storage options. Finally, our study suggests that in conducting participatory on-farm research trials, it is important to include novel treatments suggested by farmers – such as the inclusion of neem – as a benchmark from which they can compare among their own traditional and alternative options, as also suggested by van Asten et al. (2009).

## 5 Conclusions

This study aimed to elucidate farmers’ wheat seed storage practices obtained from a household survey, and to use the data collected during surveys to co-design and implement participatory farm household trials with farming households comparing hermetic SuperGrainbags® and LDPE bags storage, both with and without neem. Our results suggest that SuperGrainbags® are more effective in maintaining seed moisture levels close to pre-storage conditions and in increasing germination and coleoptile length than LDPE bags, with no effect of neem. Hence, SuperGrainbags® could potentially lend additional agronomic advantages due to rapid coleoptile expansion that could aid in wheat seedling emergence and establishment, though this hypothesis requires confirmation under field trial conditions.

SuperGrainbags® were also more effective in reducing insect damage to seed during storage. Inclusion of neem with LDPE bags also had statistically significant damage abatement effects, although at levels lower than those associated with SuperGrainbags® with or without neem. Hermetic storage bags suppressed the primary seed-predating arthropod pest of Coleoptera order with Curculionidae as the most abundant family, and weevils and other families of beetles. Where farmers used LDPE bags, neem inclusion had some additional pest suppressive effect, with similar patterns for damaged seed and the blackspot *Fusarium graminearum*. For these reasons, where farmers are unable to access or afford hermetic storage bags, the indigenous technique of applying dried neem leaves appears to confer some, albeit limited benefits for pest control. Conversely, the comparatively better and more consistent performance of SuperGrainbags® for the range of parameters and higher preference rankings by participating farmers, suggests that agricultural development programs advising wheat farmers on storage techniques in Bangladesh may wish to focus more on making hermetic storage options widely available and affordable. Lastly, this study demonstrated the value of participatory approaches that actively consult and involve farmers in the selection and design of experimental treatments, and the implementation of long-duration storage trials within their own homes. Such participatory approaches can help validate the performance of promising storage options under real-world, and hence highly relevant conditions, while also serving as a pedagogic tool for extension services and farmers alike.

## Author statement

Conceptualization: TJK, MMU. Methodology: TJK, MEB, MKG. Formal Analysis: TJK, KH. Investigation: TJK, MMU, MEB, MZK. Data curation: KH, MZH, Writing-Original Draft: TJK, JT. Writing – Review & Editing: TJK, KH, MZH, JT. Project Administration: TJK, MMU. Supervision: TJK, MKG, MMU. Funding acquisition: TJK.

## Declaration of competing interest

The authors declare that they have no known competing financial interests or personal relationships that could have appeared to influence the work reported in this paper.
